# Factors Influencing the Severity of Urinary and Defecatory Dysfunction Among the Middle-Aged and Older Adult Chinese Population: Longitudinal Study of a 5-Wave Survey Cohort

**DOI:** 10.2196/70541

**Published:** 2025-05-26

**Authors:** Haoran Zhu, Xiaoming Li, Liwei Jing, Jingli Kou, Lichao Gong, Rui Wang, Guangtian Liu, Chao Zhang, Jiayi Zhao, Jing Zhang

**Affiliations:** 1 School of Nursing, Capital Medical University, 10 Xitoutiao, You'anmenwai, Fengtai District, Beijing, 100069, China, 86 13021000866; 2 School of Public Health, North China University of Science and Technology, Tangshan, China; 3 Department of Geriatrics, Xuanwu Hospital of Capital Medical University, Beijing, China; 4 College of Nursing and Rehabilitation, North China University of Science and Technology, Tangshan, China; 5 Department of Gastroenterology, Peking University Third Hospital, Beijing, China; 6 St Michael College, University of Toronto, Toronto, ON, Canada; 7 School of Public Health, Harbin Medical University, Harbin, China

**Keywords:** China, characteristic, risk factors, urination and defecatory dysfunction, longitudinal cohort study

## Abstract

**Background:**

Urinary and defecatory dysfunction (UDD) is a significant concern among the aging population in China. However, there is a lack of longitudinal research exploring the risk factors of UDD severity in Chinese older adults.

**Objective:**

This study uses data from the China Health and Retirement Longitudinal Study spanning 2011 to 2020 to explore UDD risk factors in the middle-aged and older adult Chinese population, focusing on epidemiological characteristics and potential influences on severity.

**Methods:**

A longitudinal cohort of over 10,000 participants from the China Health and Retirement Longitudinal Study was analyzed across 5 waves using Bayesian logistic regression. This analysis examined associations between UDD severity and factors including demographic, lifestyle, and health-related factors, including comorbidities, BMI, and handgrip strength.

**Results:**

Higher UDD prevalence was observed among female population, older adults, those with low education levels, and rural residents. Depression, arthritis, and low handgrip strength emerged as critical predictors of severe UDD. Additionally, abnormal BMI, both underweight (odds ratio [OR] 3.019, 95% CI 1.484‐5.951; *P*=0.002) and obesity (OR 2.697, 95% CI 1.338‐5.217; *P*=0.005), was strongly linked to increased severity and persistence of UDD. Participants aged 66 years and older exhibited the highest UDD prevalence, with both underweight and obese individuals facing the greatest risk of persistent and worsening symptoms.

**Conclusions:**

This study is the first to longitudinally examine the risk factors of UDD severity in China’s middle-aging and aging population. The findings underscore the need for targeted interventions focusing on muscle strength rehabilitation and comorbidity management to mitigate UDD progression, contributing to improved quality of life for older individuals.

## Introduction

Population aging has become a public health issue, imposing a major global challenge across countries in recent decades [1]. The population aged 60 years and older in China is projected to exceed 300 million by 2025, accounting for more than 20% of the total population [2]. Due to the decline in muscle function and disorders of the nervous system among older people, particularly hormonal changes in female population that lead to muscle changes, and urethral sphincter atrophy in male population, millions experience urinary and defecatory dysfunction (UDD) [3-7].

The World Health Organization’s integrated care for older people model emphasizes the significance of UDD, particularly urinary incontinence, highlighting its impact on health systems worldwide. Additionally, the care burden associated with urinary retention, fecal incontinence, and constipation is substantial, underscoring the need for comprehensive strategies to address these pervasive health issues [8]. UDD is defined as a disorder that affects the storage or elimination of urine and feces [9]. Recent studies on UDD reveal varying prevalence rates. Research shows 37.1% global prevalence for female urinary incontinence [10], 18.9% for constipation in older adults [11], and 8% for fecal incontinence [12]. However, these studies, mainly from China, are limited by small, regional samples and a lack of comprehensive data on influencing factors, highlighting the need for broader research to fully understand UDD’s impact. This common disorder can have a negative impact on the quality of life of patients and exacerbate their economic burden, particularly in long-term or severe cases [13-16].

Numerous studies have demonstrated that UDD may be associated with chronic conditions (such as hypertension, diabetes, stroke, depression, and so on), abnormal BMI (overweight, obesity, or underweight), handgrip strength, and unhealthy lifestyle factors such as smoking and drinking [10,17-22]. Due to limitations in existing cross-sectional studies, which cannot confirm causal relationships, we established a cohort of over 10,000 participants, with an innovative focus on UDD severity, to study the factors influencing its severity.

The study aimed to use data from a 5-wave survey conducted by the China Health and Retirement Longitudinal Study (CHARLS) [23] to (1) describe the characteristics of UDD from 2011 to 2020 and (2) to identify the risk factors impacting the severity of UDD in China.

## Methods

### Study Design and Participants

The CHARLS, a nationally representative survey targeting individuals aged 45 years and older from 450 villages or communities in 28 provinces and 150 counties or districts across Mainland China, was conducted by the National School of Development at Peking University from 2011 to 2020 [23]. A total of 150 counties were randomly selected, with stratification by region and urban or rural classification. Administrative villages and neighborhoods were designated as primary sampling units. Households with at least 1 member aged 45 years or older were selected, and 1 individual, along with their spouse, was interviewed. The CHARLS database encompasses a broad array of variables related to demographic statistics, socioeconomic conditions, and health status. Participants in CHARLS are interviewed face-to-face every 2 years using a computer-assisted personal interview technique.

To map the characteristics and temporal trends in UDD prevalence, all participants with UDD were identified in each wave of the cross-sectional survey from 2011 to 2020. For identification of risk factors, participants with UDD who enrolled in 2011 and were continuously followed up in the subsequent 4 cross-sectional surveys were included. Detailed information about the sampling design used in the survey has been described in earlier publications [24].

### Ethical Considerations

The CHARLS was approved by the Biomedical Ethics Review Committee of Peking University (IRB0000105211015) [23]. All participants provided signed informed consent forms and received modest financial compensation. Data were anonymized before release and are only available to approved researchers.

### Data Collection

In each county or district, trained staff collected data at participants’ homes and local community health centers as well as County Centers for Disease Prevention and Control in accordance with the reported protocol [23]. Deidentified information was collected for analysis, consisting of data on participants’ demographic characteristics, health-related behaviors and outcomes, childhood conditions, community environment, cognitive and physical health, current economic status, social and family support, health insurance, health care use, comorbidities (chronic diseases such as hypertension and diabetes), and their urinary and defecation control abilities.

Referring to the World Health Organization standard, participants were divided into the following age groups: 45‐59 years (middle-aged), 60‐74 years (young-old), 75‐89 years (old), and ≥90 years (very old) [25]. Residences of participants included rural and urban areas. Education levels were categorized as illiterate, primary school graduate, secondary school graduate, and college graduate or above. Regions of China were classified as Southwest, Southern, Eastern, Northwest, Northern, and Northeast.

The CHARLS did not include data from Ningxia Hui Autonomous Region, Xizang Autonomous Region, Hainan province, Hong Kong, Macau, and Taiwan province. Additionally, the 2020 follow-up survey in the CHARLS did not include participants from the Xinjiang Uygur Autonomous Region due to the impact of the COVID-19 pandemic.

### Case Identification and Study Outcomes

According to the International Classification of Functioning, Disability, and Health, urinary function refers to the ability to discharge urine from the bladder, while bowel function refers to the ability to expel waste materials and undigested food in the form of feces, along with the associated physiological processes [26]. Therefore, impairment in either of these functions is classified as UDD. Based on participants’ responses to the question “Do you have any difficulties with controlling urination and defecation?” in CHARLS, all participants reporting “I have difficulty” or “I cannot do it” were identified as having UDD.

The second aim of the study was to explore the risk factors for UDD. To this end, participants with UDD were divided into the 4 subgroups based on clinical expert experience and the actual disease status of the participants, each comprising, individuals with increasingly severe UDD, from group A to group D, in order, as follows:

- Group A: Participants who had never experienced UDD.
- Group B: Participants who identified as having UDD in 1 or 2 consecutive surveys, without recurrence in the later follow-ups.
- Group C: Participants who reported having experienced UDD in 1 or 2 consecutive surveys and had recovered at the time of the subsequent 1 or 2 follow-ups but eventually experienced recurrence.
- Group D: Participants who identified as having UDD in consecutive 3 or more surveys.

### Statistical Analysis

To achieve the first study aim, a descriptive analysis was conducted to examine the characteristic of prevalence of UDD by sex, residence, marital status, age group, education level, and geographic region over 5 waves from 2011 to 2020. The calculation of the prevalence rate is the cumulative number of cases divided by the total number of participants followed up per year.

To achieve the second study aim, a chi-square test was initially performed to identify potential risk factors among the 4 groups. These factors were then further examined through Bayesian logistic regression analysis. Prior to regression analysis, chi-square tests were conducted to assess univariate associations. A weakly informative prior was used to ensure robust parameter estimation. Parameter estimation was conducted using Markov Chain Monte Carlo sampling, and model results are presented as odds ratios (ORs) with 95% CIs. To enhance interpretability, exponentiation was applied to the estimated coefficients, and 2-sided *P* values were calculated. R (version 64 4.4.3; R Foundation for Statistical Computing) was used for descriptive analysis and logistic regression. *P* values were 2-sided with a significance level of .05.

## Results

### Characteristics of Study Participants in the 5 Waves

**Figure 1.**
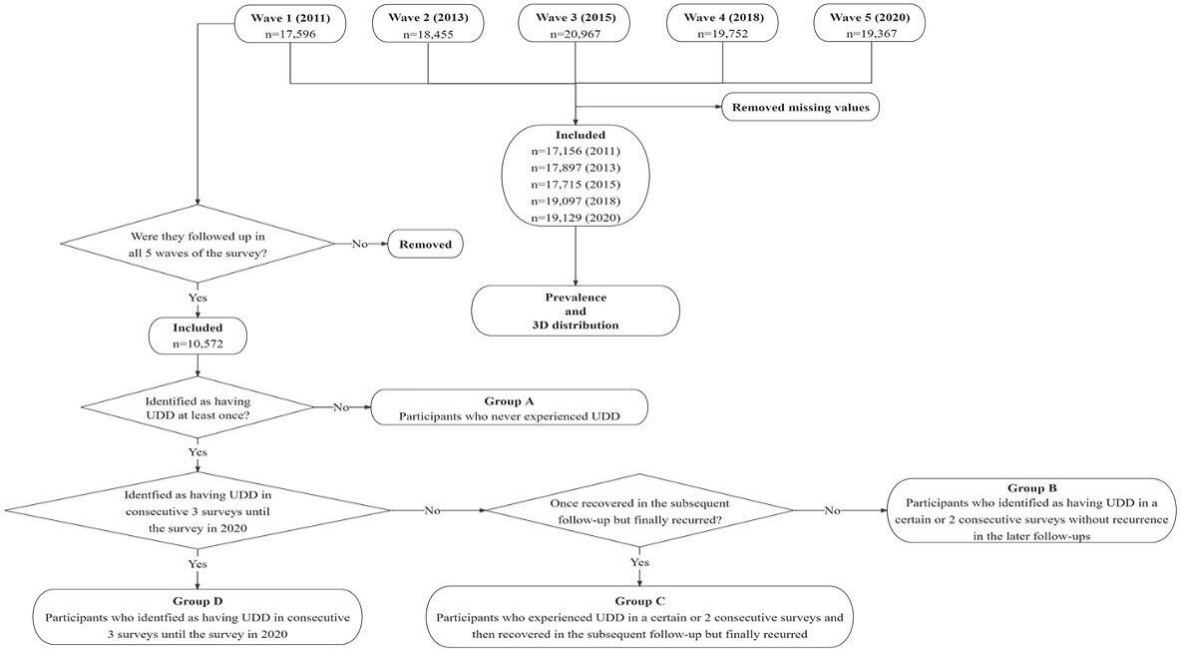
Flowchart of participant inclusion or exclusion and grouping diagram*.* UDD: urinary and defecatory dysfunction.

The participants in the 5 waves comprised 17,156, 17,897, 17,715, 19,097, and 19,129 individuals, respectively. Although there were no significant differences between sexes, significant differences were found among age groups, places of residence, education levels, marital status, and regions across the 5 waves. Figure 1 shows the screening process of this study. Table 1 presents the characteristics of patients with UDD across various groups.

**Table 1.** Demographic characteristics of participants with urinary and defecatory dysfunction in the 5-wave survey from 2011 to 2020.

	Baseline wave in 2011 (n=17,156), n (%)	Second wave in 2013 (n=17,897), n (%)	Third wave in 2015 (n=17,715), n (%)	Fourth wave in 2018 (n=19,097), n (%)	Fifth wave in 2020 (n=19,129), n (%)	*P* value
Sex						.98
Female	388 (4.41)	349 (3.77)	429 (4.66)	491 (4.89)	513 (5.09)	
Male	365 (4.63)	372 (4.31)	401 (4.71)	411 (4.54)	447 (4.94)	
Age group (years)						<.001
45‐50	57 (1.49)	54 (1.46)	47 (1.62)	31 (1.30)	32 (1.27)	
51‐55	59 (2.11)	58 (2.18)	76 (2.43)	54 (1.47)	73 (1.97)	
56‐60	130 (3.72)	95 (2.75)	97 (3.34)	67 (2.48)	90 (3.21)	
61‐65	132 (4.91)	133 (4.31)	136 (4.19)	132 (3.87)	161 (4.69)	
66‐70	106 (5.92)	104 (5.15)	138 (5.99)	185 (6.24)	199 (6.66)	
71‐75	92 (7.55)	110 (7.71)	129 (8.40)	134 (7.49)	154 (8.75)	
76‐80	67 (8.54)	80 (8.71)	111 (11.28)	144 (12.04)	122 (11.37)	
≥81	110 (20.04)	87 (13.30)	96 (13.64)	155 (15.78)	129 (15.64)	
Education						<.001
Illiterate^a^	476 (6.15)	414 (5.09)	482 (5.99)	545 (6.48)	537 (6.51)	
Primary^b^	146 (3.98)	147 (3.88)	177 (4.59)	185 (4.42)	205 (4.90)	
Second^c^	122 (2.29)	150 (2.70)	162 (3)	162 (2.64)	182 (3.13)	
College^d^	9 (2.14)	10 (2.43)	9 (2.17)	10 (2.63)	36 (4.17)	
Marital status						<.001
Married^e^	593 (3.96)	560 (3.61)	625 (4.09)	635 (3.92)	695 (4.34)	
Other status^f^	160 (7.30)	161 (6.78)	205 (8.37)	267 (9.19)	265 (8.51)	
Residence						<.001
Rural	627 (4.72)	585 (4.27)	637 (4.70)	738 (4.87)	728 (5.11)	
City	122 (3.24)	131 (3.27)	180 (4.64)	163 (4.17)	143 (4.99)	
Region						<.001
Northeast	66 (5.18)	52 (3.75)	59 (4.52)	68 (5.16)	64 (5.41)	
East	222 (4.27)	159 (2.93)	201 (3.63)	213 (3.62)	263 (4.38)	
North	87 (3.72)	124 (5.13)	119 (5.19)	146 (5.75)	136 (5.64)	
Central	94 (3.49)	119 (4.26)	126 (4.61)	150 (5.11)	162 (5.32)	
South	42 (2.76)	49 (3.25)	64 (4.37)	58 (3.66)	78 (4.75)	
Southwest	199 (6.84)	163 (5.25)	217 (7.04)	204 (6.06)	185 (5.39)	
Northwest	43 (3.51)	55 (4.40)	44 (3.39)	63 (4.29)	72 (5.18)	

a Without formal education, did not finish primary school, was homeschooled.

b With primary school education.

c With middle school or high school education.

d With college education and above.

e Married with spouse present or married but not living with spouse temporarily for reasons such as work.

f Separated, divorced, widowed, or never married.

### Trends and Distributions of UDD Prevalence From 2011 to 2020

**Figure 2.**
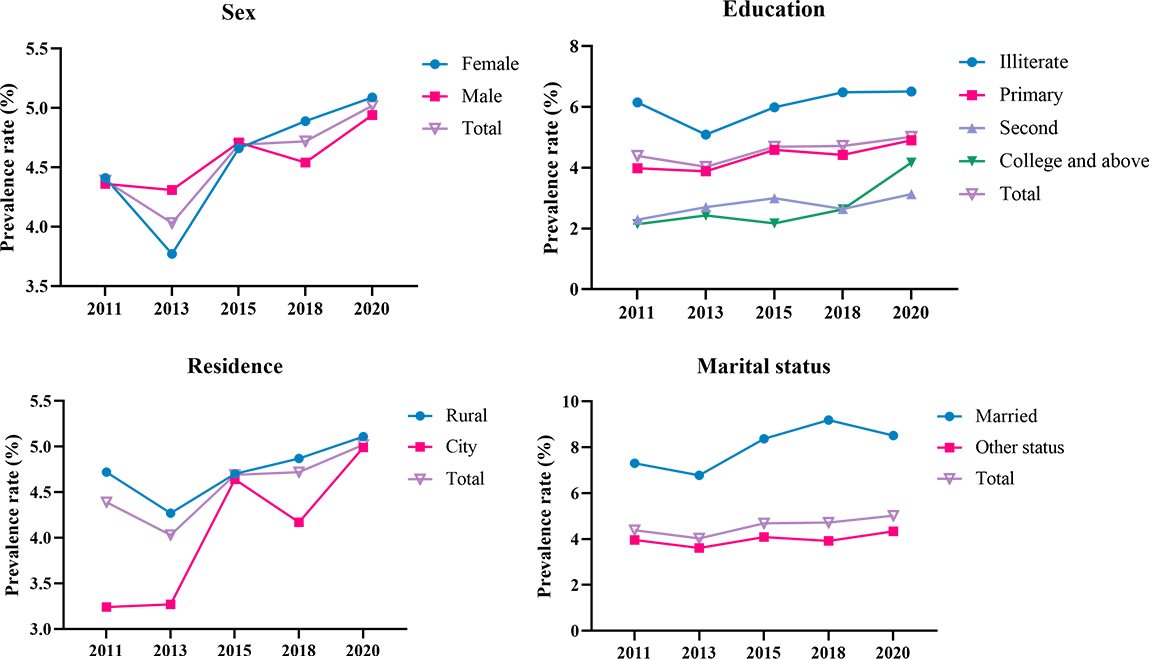
Temporal trends of prevalence of urinary and defecatory dysfunction by sex, education level, marital status, and residence among middle-aged and older people in China from 2011 to 2020.

The prevalence of UDD from 2011 to 2020 was 4.39% (n=753), 4.03% (n=721), 4.69% (n=830), 4.72% (n=902), and 5.02% (n=960), respectively. Figure 2 demonstrates that the prevalence of UDD by sex and residence rose slowly over the 10 years, with female participants or participants living in rural areas having higher prevalence than their counterparts. The prevalence of UDD by education level and marital status remained essentially stable across the decade in China, although illiterate or married participants had higher prevalence than other categories.

**Figure 3.**
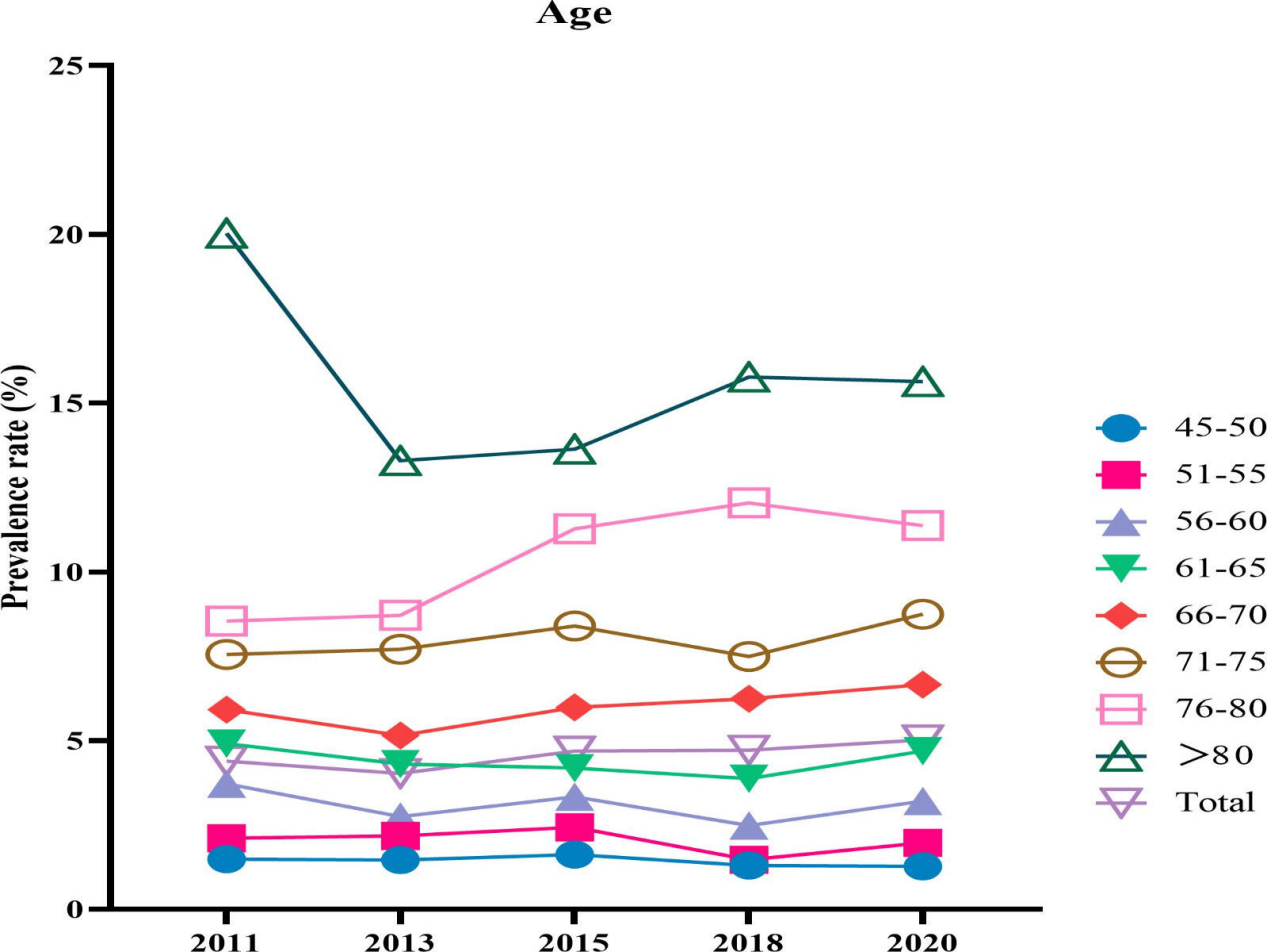
Temporal trends of age-specific prevalence (in years) of urinary and defecatory dysfunction among middle-aged and older people in China from 2011 to 2020.

Figure 3 illustrates that the prevalence of UDD gradually increased with age over the decade, particularly among those aged 66 years and older who had a higher prevalence than the overall prevalence. The prevalence of the 4 groups aged 66 years and older slightly increased, while the corresponding figures for the 4 younger groups younger than 65 years were almost stable during the 10 years of surveys.

**Figure 4.**
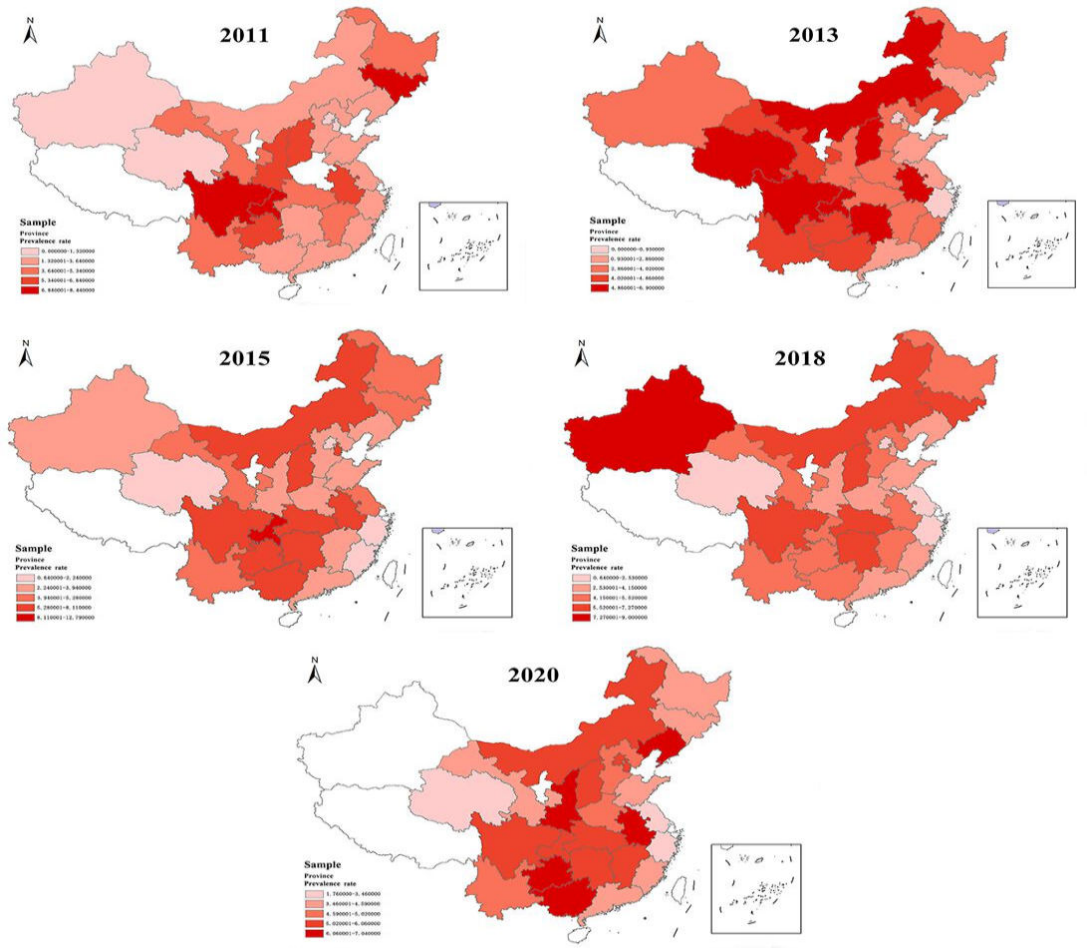
Regional distribution of urinary and defecatory dysfunction prevalence among middle-aged and older people in China from 2011 to 2020 (because of the impact of the pandemic, information from Xinjiang participants in 2020 was not included in the China Health and Retirement Longitudinal Study database).

The prevalence of UDD varied significantly across different regions over the years, being consistently higher in the Southwest region than in other regions. Both the Southwest and Northwest regions saw an increasing prevalence of UDD in the past 10 years (Figure 4).

### Identification of Risk Factors for Different UDD Conditions in a Cohort Study

There were 10,572 participants enrolled in the cohort study; among them, 8966 participants had never experienced UDD over the 10-year period, while 1275 had recovered, 245 reported recurrence, and 86 reported persistence at the last follow-up. There were significant differences for factors other than sex (*P*=.19), cancer (*P*=.12), and smoking (*P*=.39). Among all groups with various severity of UDD, the proportion of female participants was slightly higher than that of male participants. Higher proportions of participants were aged 60‐75 years, illiterate, married, living in rural areas, living in the Southwest and East regions, with normal BMI, nonsmokers, and nondrinkers. The comorbidities of participants with UDD are shown in Table 2.

**Table 2.** Univariable analysis of 4 groups of urinary and defecatory dysfunction (UDD).

	Without UDD^a^ (group A; n=8966), n (%)	Recovered from UDD^b^ (group B; n=1275), n (%)	Reported recurrent^c^ UDD (group C; n=245), n (%)	Reported persistent UDD^d^ (group D; n=86), n (%)	*P* value^e^
Sex					
Female	4751 (52.99)	717 (56.24)	132 (53.88)	47 (54.65）	.19
Age group (years)					<.001
45‐59	5604 (62.50)	555 (43.53)	85 (34.69)	30 (34.88)	
60‐75	3015 (33.63)	608 (47.69)	137 (55.92)	47 (54.65)	
76‐90	343 (3.83)	109 (8.55)	22 (8.98)	8 (9.30)	
＞90	4 (0.04)	3 (0.24)	1 (0.41)	1 (1.16)	
Education					<.001
Illiterate	3935 (43.89)	715 (56.08)	147 (60)	47 (54.65)	
Primary	1967 (21.94)	289 (22.67)	46 (18.78)	26 (30.23)	
Second	2918 (32.55)	260 (20.39)	50 (20.41)	12 (13.95)	
College and above	146 (1.63)	11 (0.86)	2 (0.82)	1 (1.16)	
Marital status					
Married	8169 (91.11)	1088 (85.33)	210 (85.71)	74 (86.05)	<.001
Residence					
Rural	7400 (82.53)	1115 (87.45)	213 (86.94)	74 (86.05)	.002
Region					<.001
Northeast	557 (6.21)	75 (5.88)	15 (6.12)	6 (6.98)	
East	2891 (32.24)	351 (27.53)	63 (25.71)	22 (25.58)	
North	1149 (12.82)	183 (14.35)	30 (12.24)	11 (12.79)	
Central	1482 (16.53)	215 (16.86)	40 (16.33)	14 (16.28)	
South	735 (8.20)	84 (6.59)	13 (5.31)	4 (4.65)	
Southwest	1521 (16.96)	279 (21.88)	68 (27.76)	26 (30.23)	
Northwest	631 (7.04)	88 (6.90)	16 (6.53)	3 (3.49)	
BMI^e^					<.001
Underweight	466 (5.41)	83 (6.83)	24 (10.57)	14 (17.07)	
Normal weight	4486 (52.09)	636 (52.35)	113 (49.78)	29 (35.37)	
Overweight	2615 (30.36)	330 (27.16)	64 (28.19)	20 (24.39)	
Obese	1018 (11.82)	157 (12.92)	25 (11.01)	17 (20.73)	
Smoking^e^	3434 (38.32)	483 (37.97)	107 (43.67)	33 (38.37)	.39
Drinking^e^	3065 (34.21)	342 (26.89)	83 (33.88)	25 (29.07)	<.001
Comorbidities					
Hypertension^e^	1882 (21.09)	387 (30.57)	71 (29.22)	32 (37.21)	<.001
Dyslipidemia^e^	772 (8.64)	144 (11.31)	30 (12.35)	11 (12.79)	.002
Diabetes^e^	424 (4.73)	83 (6.52)	22 (9.05)	10 (11.63)	<.001
Cancer	76 (0.85)	19 (1.49)	1 (0.41)	1 (1.16)	.12
Chronic lung disease^e^	701 (7.84)	184 (14.51)	40 (16.39)	20 (23.26)	<.001
Liver disease^e^	320 (3.57)	59 (4.63)	16 (6.53)	3 (3.49)	.03
Heart problems^e^	854 (9.53)	210 (16.51)	45 (18.44)	18 (21.18)	<.001
Stroke^e^	125 (1.40)	41 (3.24)	12 (4.92)	6 (7.06)	<.001
Kidney disease^e^	472 (5.27)	110 (8.63)	35 (14.29)	14 (16.28)	<.001
Digestive disease^e^	1954 (21.86)	370 (29.20)	96 (39.18)	37 (43.53)	<.001
Memory-related disease^e^	66 (0.74)	26 (2.05)	12 (4.90)	6 (6.98)	<.001
Arthritis or rheumatism^e^	2839 (31.74)	578 (45.62)	141 (57.55)	50 (58.14)	<.001
Asthma^e^	239 (2.68)	53 (4.18)	24 (9.84)	6 (6.98)	<.001
Depression^e^	3334 (37.18)	748 (58.67)	174 (71.02)	65 (75.58)	<.001
Handgrip strength					
Low handgrip strength	1005 (11.84)	289 (24.41)	70 (31.39)	30 (37.04)	<.001

a Participants who had never experienced UDD.

b Participants who identified as having UDD in 1 or 2 consecutive surveys without recurrence in the later follow-ups.

c Participants who reported experiencing UDD in 1 or 2 consecutive surveys and having recovered in the subsequent 1 or 2 follow-ups but eventually experienced recurrence.

d Participants who identified as having UDD in 3 or more consecutive surveys.

e Missing data: 436 for BMI, 7 for smoking, 10 for drinking, 53 for hypertension, 30 for dyslipidemia, 14 for diabetes, 27 for chronic lung disease, 4 for liver disease, 13 for heart problem, 36 for stroke, 5 for kidney disease, 37 for digestive disease, 42 for memory-related disease, 29 for arthritis or rheumatism, 52 for asthma, and 593 for handgrip strength.

**Figure 5.**
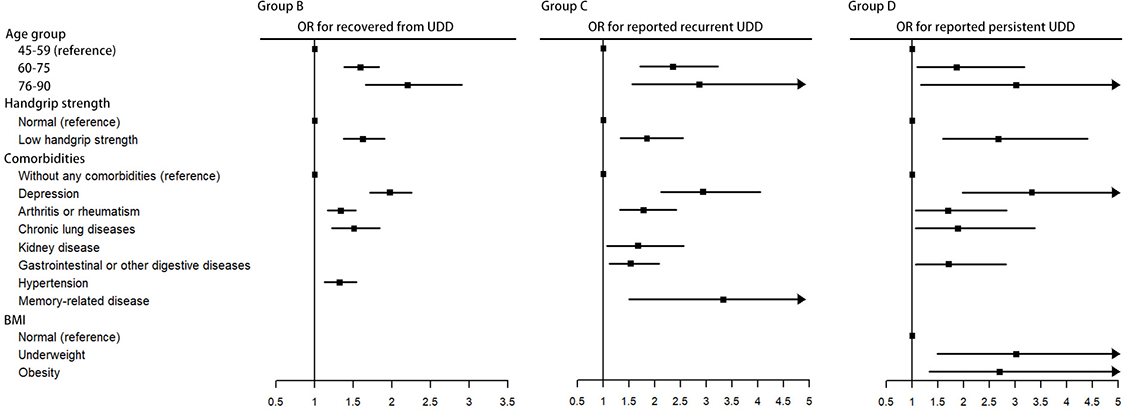
OR and 95% CI of risk factors for UDD in different population groups with UDD. Forest plots show ORs and 95% CIs for group (B) recovered from UDD, (C) reported UDD recurrence, and (D) UDD persisted adjusted for sex, age, education, marital status, residence, region, BMI, handgrip strength, smoking status, drinking status, and comorbidities. In the forest plot, only the significant results within each group (*P*<.05) are shown. The reference group for groups B, C, and D consists of participants who had never experienced UDD. OR: odds ratio; UDD: urinary and defecatory dysfunction.

Figure 5 shows the ORs and 95% CIs of the risk factors for UDD in patients with different conditions. Four common risk factors were identified across the 3 groups, namely, older age (60‐89 years), depression, arthritis (or rheumatism), and handgrip strength. For group C and group D, who had more severe UDD than group B, participants with gastrointestinal diseases had a higher risk of experiencing recurrent or persistent UDD. Memory-related diseases (OR 3.328, 95% CI 1.505‐6.836; *P*=.002) may cause UDD to recur. Additionally, underweight (OR 3.019, 95% CI 1.484‐5.951; *P*=.002) and obesity (OR 2.697, 95% CI 1.338‐5.217; *P=.*005) were identified as potential independent risk factors for the persistent nature of UDD among middle-aged and older people in China. The detailed results of the Bayesian logistic regression model are provided in Multimedia Appendix 1.

## Discussion

### Principal Findings

This is the first study to use a representative sample to describe the temporal, spatial, and interpersonal distributions of UDD among middle-aged and older people in China. The study identified that over the past decade, the prevalence of UDD has remained stable across different education levels, kinds of marital status, and age groups. However, it significantly increased among both sexes, urban residents, and in the Northwest and Southwest regions. Participants who were female, illiterate, or married and those aged 66 years and older exhibited a higher prevalence of UDD. The incidence, recurrence, and persistence of UDD were more likely to be influenced by aging and comorbidities such as depression, arthritis (or rheumatic diseases), and handgrip strength in China. More importantly, being underweight or obese might contribute to the long-term persistence of UDD.

### The Prevalence and Time Trends of UDD

Due to its increased prevalence, the disease burden associated with UDD has shown a gradually escalating trend in China over the past 10 years. However, despite a slight increase, the prevalence of UDD in China (5.02% in 2020) has remained lower than the global prevalence, which ranges from 7% to 55%. This lower prevalence might be attributed to lower self-report due to participants’ potential embarrassment or their belief that UDD is a natural aging-related phenomenon among the Chinese population [10,21,27,28]. Approximately, 50% to 67% of patients were unwilling to report their UDD conditions to health care providers [29,30]. The slowly increasing trend in prevalence observed in this study may reflect improved awareness and diagnosis of UDD over time, leading to more UDD cases being identified.

Over the past decade, the prevalence of UDD in female individuals has consistently remained higher than that in male individuals. This finding underscores the necessity of addressing the unique physiological and reproductive health challenges that female individuals face, as the relaxation of pelvic floor muscles during childbirth significantly contributes to this increased prevalence [31]. This suggests that we should place greater emphasis on female individual’s pelvic health. By implementing early interventions and training, such as pelvic floor muscle exercises, we can significantly improve the quality of life for female individuals in their later years [32].

It was notable that the Northwest and Southwest provinces—2 resource-limited regions in China—experienced an obviously higher prevalence of UDD during the 5-wave survey. The evidence aligned with other studies demonstrating that economic status may influence the prevalence of UDD, while UDD places a significant economic burden on patients too [14,15,33]. The higher prevalence of UDD among participants from resource-limited areas may be related to the insufficient health insurance or poor access to medical services, such as postpartum pelvic floor rehabilitation [34,35]. Therefore, economic development may serve as a solution to improve the quality of life of patients with UDD in these regions along with enhancing the accessibility of medical resources.

Given the impact of UDD, it is necessary to establish a surveillance system for UDD to provide more evidence for the identification of the long-term impact of UDD and the development of prevention and treatment strategies at the population level.

### Population Aging, Comorbidities, and Low Handgrip Strength May Lead to All Severity of UDD Among the Chinese Population as Marked Risk Factors

Population aging is an important risk factor influencing the occurrence, recurrence, and persistence of UDD. This study suggests that the physiological senescence associated with aging may contribute to the development and exacerbation of UDD, particularly among participants aged 66-90 years. It is widely recognized that aging can lead to the occurrence or aggravation of UDD, likely due to the frailty and functional impairment associated with aging [13,36-39]. However, the potential mechanism underlying the association between frailty and UDD remains unknown. Finite element models can make it possible to explore the mechanism underpinning the relationship between frailty and UDD [40]. By comparing defecation outcomes under different parameter settings, the association between training methods and urinary and fecal control ability can be quantified, providing accurate guidance for rehabilitation.

This study demonstrated that comorbidities are the second important risk factor for UDD. For instance, depression was found to significantly impact the incidence, recurrence, and persistence of UDD, as it was usually associated with reduced serotonin function, which leads to urgency urinary incontinence [41]. Some studies argue that the medicines patients take potentially result in gastrointestinal side effects such as constipation [42]. In addition, participants with urinary and fecal incontinence and constipation exhibit significantly higher levels of depression and stress [43-49], possibly due to the production of certain gut microbiota that lead to depressive symptoms and lower levels of *g_Pseudoramibacter-Eubacterium* and *g_Candidatus-Solibacter*. [50] Functional constipation in middle-aged and older individuals may lead to a decrease in the abundance of these microbiota [51], ultimately exacerbating depression. This interaction creates a vicious cycle between depression and UDD.

In addition to depression, arthritis or rheumatism likely also contributes to the increased risk of incidence, recurrence, and persistence of UDD, although its impact on UDD might have been underestimated over the past 20 years. The most recent studies on the association between arthritis and UDD were conducted in the 1990s. These studies implied that arthritis may increase susceptibility to urinary tract infections, and atlantoaxial subluxation in the late stage of arthritis may lead to neurogenic bladder [52], which in turn increases the incidence of UDD. Medications such as misoprostol and cyclophosphamide, used to treat arthritis, may also cause UDD [52,53]. Moreover, opioids, commonly taken by patients with arthritis and rheumatic diseases, are likely to cause constipation. Additionally, the limited mobility of patients with osteoarthritis is perhaps associated with urinary incontinence [54,55].

Other comorbidities, such as hypertension and memory-related diseases, were also identified as potential contributors to the occurrence or recurrence of UDD. For instance, studies indicate a correlation between hypertension and constipation, potentially linked by shared physiological mechanisms and lifestyle factors [56]. Patients with Alzheimer disease often lose the ability to send bladder signals to the brain’s urination center, resulting in an inability to urinate normally [57,58].

This study, therefore, suggested potential causal relationships between comorbidities and UDD, indicating that health care providers should pay more attention to the prevention and treatment of these comorbidities. Optimizing pharmaceutical therapy may effectively alleviate the incidence and progression of UDD in middle-aged and older individuals. Additionally, increasing physical therapy or dietary and nutritional treatments for mental health, arthritis or rheumatic diseases, and other chronic conditions could be beneficial. Dissemination of knowledge about these chronic diseases to enhance public awareness at the community level might help address the occurrence, recurrence, and persistence of UDD.

Our study also reveals a significant correlation between grip strength and the severity of UDD. Specifically, individuals with reduced grip strength exhibited a higher incidence of severe UDD manifestations, including recurrent and persistent conditions. This relationship may suggest that grip strength serves as an indirect biomarker for overall muscle integrity and functional capacity, which could influence the severity of UDD symptoms [59,60]. These results align with existing literature emphasizing the importance of muscle function in maintaining urogenital health and also underscore the need for further research into preventive strategies focusing on strength training in vulnerable populations.

### Being Underweight or Obese Can Lead to Long-Term Persistence of UDD

This study found that almost half of the older Chinese population in the cohort had abnormal weight, with underweight or obesity considered independent risk factors for persistent UDD. This finding aligned with evidence from other studies showing that obesity is closely related to the occurrence of UDD [10,61-63]. The mechanism underlying this association is that metabolic changes and increased abdominal pressure caused by obesity may lead to the development of UDD. Additionally, underweight older adults might experience reduced muscle strength, particularly in the pelvic floor, possibly triggering the incidence of UDD. Some studies argue that population aging–related muscle atrophy and decreased strength are more likely to cause persistence of UDD [64,65], particularly among those who are underweight. Consequently, the improvement of nutritional intake is vital for older people in both communities and health care facilities. The awareness of healthy diet and appropriate physical exercises be enhanced among the older population, and health care providers and community health workers should develop individual tailored interventions for patients with UDD who are underweight or obese.

### Strengths and Limitations

The study population was selected from 28 provinces in China, ensuring sufficient representativeness to reflect the characteristics and risk factors of UDD in the middle-aged and older Chinese populations. To achieve the second study objective, a cohort study was designed to establish clear causal relationships between the identified risk factors and the incidence, progression, and persistence of UDD in the Chinese population. Additionally, the population with UDD was classified based on the severity of UDD, facilitating the exploration of risk factors and the development of personalized interventions.

There are some limitations to this study. The CHARLS does not provide detailed information on urinary and fecal incontinence or constipation, making it difficult to distinguish risk factors for diverse UDD conditions. The CHARLS also lacks information on factors that may affect UDD, such as bladder outlet obstruction, urinary tract infections, benign prostatic hyperplasia, use of analgesics, and history of abuse, and factors such as hospital capacity, network scale, and accessibility of medical services over the past decade could not be analyzed due to the same limitations. Additionally, due to the statistical methods used in the CHARLS database, the impact of incontinence related to childbirth in female population may have been underestimated. Consequently, this study could not assess the impact of these factors on UDD. Furthermore, those who had UDD in both 2018 and 2020 could not have their UDD prognosis determined after the CHARLS survey: they may have recovered and not be included in the most-severe group, which may have resulted in misclassification bias. In addition, the emergence of recovered participants may also be related to respondents’ misjudgment of their own UDD conditions. In the Results section, middle school and high school education emerged as a protective factor for the recovered population, though the underlying reasons require further investigation.

### Conclusions

In summary, the prevalence of UDD increased with age and was found to be higher among illiterate individuals, married people, and those living in the Southwest and Northwest regions. Depression, arthritis and rheumatic diseases, and other chronic comorbidities contributed to the occurrence, recurrence, and persistence of UDD; further, being underweight or obese independently affected the persistence of UDD among the middle-aged and older Chinese population. Enhancing the treatment of psychological and chronic diseases and improving BMI may alleviate the occurrence, recurrence, and persistence of UDD.

## Supplementary material

10.2196/70541 Multimedia Appendix 1 The detailed results of the Bayesian logistic regression model.

## Abbreviations

CHARLS: China Health and Retirement Longitudinal Study
OR: odds ratio
UDD: urinary and defecatory dysfunction
