# Supplementary material for: Factors Influencing the Severity of Urinary and Defecatory Dysfunction Among the Middle-Aged and Older Adult Chinese Population: Longitudinal Study of a 5-Wave Survey Cohort
Source: JMIR Aging. 2025 May 26;8:e70541. doi: 10.2196/70541 (PMC12129371; doi:10.2196/70541)
Supplement: Multimedia Appendix 1 [file aging-v8-e70541-s001.docx]

|  | Recovered from UDD  (Group B)  N=1,275 | | | Reported recurrent  UDD (Group C)  N=245 | | | Reported persistent UDD  (Group D)  N=86 | | |
| --- | --- | --- | --- | --- | --- | --- | --- | --- | --- |
|  | OR | 95% CI | *P* value | OR | 95% CI | *P* value | OR | 95% CI | *P* value |
| Age group |  |  |  |  |  |  |  |  |  |
| 45–59 | Reference | Reference | Reference | Reference | Reference | Reference | Reference | Reference | Reference |
| 60–75 | 1.589 | 1.377-1.831 | ＜0.001 | 2.349 | 1.711-3.224 | ＜0.001 | 1.861 | 1.096-3.175 | 0.021 |
| 76–90 | 2.203 | 1.662-2.908 | ＜0.001 | 2.859 | 1.555-5.079 | ＜0.001 | 3.020 | 1.170-7.333 | 0.019 |
| ＞90 | 2.267 | 0.359-11.355 | 0.351 | 3.927 | 0.279-33.373 | 0.265 | 6.366 | 0.366-65.186 | 0.162 |
| Education |  |  |  |  |  |  |  |  |  |
| Illiterate | Reference | Reference | Reference | Reference | Reference | Reference | Reference | Reference | Reference |
| Primary | 0.864 | 0.731-1.019 | 0.084 | 0.766 | 0.527-1.098 | 0.161 | 1.549 | 0.886-2.668 | 0.119 |
| Second | 0.686 | 0.577-0.818 | ＜0.001 | 0.746 | 0.502-1.104 | 0.145 | 0.720 | 0.335-1.468 | 0.376 |
| College and above | 0.606 | 0.302-1.120 | 0.137 | 0.649 | 0.133-2.213 | 0.555 | 0.939 | 0.097-5.127 | 0.951 |
| Marital Status |  |  |  |  |  |  |  |  |  |
| Married | Reference | Reference | Reference | Reference | Reference | Reference | Reference | Reference | Reference |
| Other Status | 1.129 | 0.928-1.378 | 0.234 | 1.052 | 0.679-1.590 | 0.818 | 0.964 | 0.455-1.875 | 0.919 |
| Region |  |  |  |  |  |  |  |  |  |
| Northeast | Reference | Reference | Reference | Reference | Reference | Reference | Reference | Reference | Reference |
| East | 0.838 | 0.624-1.130 | 0.239 | 0.872 | 0.477-1.654 | 0.665 | 0.768 | 0.318-1.932 | 0.570 |
| North | 1.217 | 0.892-1.673 | 0.219 | 1.082 | 0.548-2.161 | 0.822 | 1.013 | 0.381-2.715 | 0.979 |
| Central | 1.049 | 0.771-1.424 | 0.759 | 1.101 | 0.591-2.112 | 0.768 | 0.898 | 0.351-2.381 | 0.824 |
| South | 0.867 | 0.606-1.240 | 0.436 | 0.865 | 0.399-1.925 | 0.720 | 0.494 | 0.121-1.855 | 0.311 |
| Southwest | 1.082 | 0.792-1.468 | 0.613 | 1.231 | 0.668-2.352 | 0.520 | 1.143 | 0.470-2.945 | 0.777 |
| Northwest | 0.824 | 0.577-1.185 | 0.296 | 0.755 | 0.348-1.644 | 0.478 | 0.424 | 0.102-1.534 | 0.218 |
| BMI ^a^ |  |  |  |  |  |  |  |  |  |
| Underweight | 0.853 | 0.646-1.119 | 0.251 | 1.381 | 0.840-2.207 | 0.189 | 3.019 | 1.484-5.951 | 0.002 |
| Normal Weight | Reference | Reference | Reference | Reference | Reference | Reference | Reference | Reference | Reference |
| Overweight | 0.944 | 0.807-1.104 | 0.468 | 1.014 | 0.713-1.429 | 0.938 | 1.382 | 0.734-2.571 | 0.308 |
| Obese | 1.049 | 0.847-1.288 | 0.654 | 1.031 | 0.620-1.665 | 0.903 | 2.697 | 1.338-5.217 | 0.005 |
| Drinking ^a^ | 0.887 | 0.766-1.026 | 0.106 | 1.270 | 0.927-1.717 | 0.129 | 1.412 | 0.831-2.362 | 0.194 |
| Comorbidities |  |  |  |  |  |  |  |  |  |
| Hypertension ^a^ | 1.319 | 1.126-1.539 | 0.001 | 1.133 | 0.803-1.590 | 0.476 | 1.438 | 0.833-2.435 | 0.187 |
| Dyslipidemia ^a^ | 1.121 | 0.893-1.398 | 0.317 | 1.183 | 0.727-1.875 | 0.489 | 0.831 | 0.365-1.737 | 0.646 |
| Diabetes ^a^ | 1.093 | 0.818-1.439 | 0.541 | 1.606 | 0.945-2.655 | 0.071 | 1.401 | 0.564-3.064 | 0.436 |
| Chronic lung disease ^a^ | 1.507 | 1.226-1.840 | ＜0.001 | 1.189 | 0.771-1.785 | 0.418 | 1.888 | 1.003-3.376 | 0.039 |
| Liver disease ^a^ | 1.052 | 0.768-1.436 | 0.754 | 1.338 | 0.740-2.300 | 0.314 | 0.588 | 0.157-1.756 | 0.393 |
| Heart problems ^a^ | 1.208 | 0.987-1.466 | 0.062 | 1.277 | 0.855-1.875 | 0.220 | 1.478 | 0.790-2.637 | 0.204 |
| Stroke ^a^ | 1.391 | 0.916-2.074 | 0.115 | 1.560 | 0.694-3.135 | 0.243 | 1.883 | 0.549-5.272 | 0.270 |
| Kidney disease ^a^ | 1.273 | 0.990-1.629 | 0.057 | 1.677 | 1.074-2.562 | 0.018 | 1.593 | 0.768-3.116 | 0.190 |
| Digestive disease ^a^ | 1.150 | 0.989-1.339 | 0.070 | 1.527 | 1.113-2.085 | 0.008 | 1.711 | 1.040-2.820 | 0.034 |
| Memory-related disease ^a^ | 1.493 | 0.857-2.533 | 0.152 | 3.328 | 1.505-6.836 | 0.002 | 2.562 | 0.610-8.365 | 0.159 |
| Arthritis or rheumatism ^a^ | 1.338 | 1.167-1.532 | ＜0.001 | 1.777 | 1.322-2.412 | ＜0.001 | 1.694 | 1.022-2.839 | 0.040 |
| Asthma ^a^ | 0.977 | 0.693-1.371 | 0.894 | 1.652 | 0.924-2.858 | 0.081 | 0.858 | 0.300-2.169 | 0.761 |
| Depression ^a^ | 1.970 | 1.718-2.257 | ＜0.001 | 2.931 | 2.119-4.051 | ＜0.001 | 3.321 | 1.972-5.776 | ＜0.001 |
| Handgrip Strength |  |  |  |  |  |  |  |  |  |
| Normal Handgrip Strength | Reference | Reference | Reference | Reference | Reference | Reference | Reference | Reference | Reference |
| Low handgrip strength | 1.620 | 1.376-1.906 | ＜0.001 | 1.846 | 1.329-2.545 | ＜0.001 | 2.673 | 1.596-4.410 | ＜0.001 |

**Note:** ***Without UDD:*** Participants who had never experienced UDD. ***Recovered from UDD:*** Participants who identified as having UDD in one or two consecutive surveys without recurrence in the later follow-ups. ***Reported recurrent UDD:*** Participants who reported experiencing UDD in one or two consecutive surveys and having recovered in the subsequent one or two follow-ups, but eventually experienced recurrence. ***Reported persistent UDD:*** Participants who identified as having UDD in three or more consecutive surveys.

BMI, body mass index.

^a^ Missing data: 436 for BMI, 7 for smoking, 10 for drinking, 53 for hypertension, 30 for dyslipidemia, 14 for diabetes, 27 for chronic lung disease, 4 for liver disease, 13 for heart problem, 36 for stroke, 5 for kidney disease, 37 for digestive disease, 42 for memory-related disease, 29 for arthritis or rheumatism, 52 for asthma, and 593 for handgrip strength.
